# A Metabolic Reprogramming Amino Acid Polymer as an Immunosurveillance Activator and Leukemia Targeting Drug Carrier for T‐Cell Acute Lymphoblastic Leukemia

**DOI:** 10.1002/advs.202104134

**Published:** 2022-01-26

**Authors:** Changzheng Li, Xinru You, Xi Xu, Binghuo Wu, Yuye Liu, Tong Tong, Jie Chen, Yishan Li, Chunlei Dai, Zhitao Ye, Xiaobin Tian, Yan Wei, Zechen Hao, Linjia Jiang, Jun Wu, Meng Zhao

**Affiliations:** ^1^ Guangdong Provincial Key Laboratory of Malignant Tumor Epigenetics and Gene Regulation, Sun Yat‐Sen Memorial Hospital Sun Yat‐sen University Guangzhou Guangdong China; ^2^ Key Laboratory of Stem Cells and Tissue Engineering (Ministry of Education) Zhongshan School of Medicine Sun Yat‐sen University Guangzhou Guangdong China; ^3^ School of Biomedical Engineering Sun Yat‐sen University Shenzhen Guangdong China; ^4^ Department of Hematology Nanfang Hospital Southern Medical University Guangzhou Guangdong China

**Keywords:** amino acid metabolism, immunosurveillance, Metabolic Reprogramming Immunosurveillance Activation Nanomedicine (MRIAN), myeloid‐derived suppressor cells (MDSCs), T‐cell acute lymphoblastic leukemia (T‐ALL)

## Abstract

Compromised immunosurveillance leads to chemotherapy resistance and disease relapse of hematological malignancies. Amino acid metabolism regulates immune responses and cancer; however, a druggable amino acid metabolite to enhance antitumor immunosurveillance and improve leukemia targeting‐therapy efficacy remains unexplored. Here, an l‐phenylalanine polymer, Metabolic Reprogramming Immunosurveillance Activation Nanomedicine (MRIAN), is invented to effectively target bone marrow (BM) and activate the immune surveillance in T‐cell acute lymphoblastic leukemia (T‐ALL) by inhibiting myeloid‐derived suppressor cells (MDSCs) in T‐ALL murine model. Stable‐isotope tracer and in vivo drug distribution experiments show that T‐ALL cells and MDSCs have enhanced cellular uptake of l‐phenylalanine and MRIANs than normal hematopoietic cells and progenitors. Therefore, MRIAN assembled Doxorubicin (MRIAN‐Dox) specifically targets T‐ALL cells and MDSCs but spare normal hematopoietic cells and hematopoietic stem and progenitor cells with enhanced leukemic elimination efficiency. Consequently, MRIAN‐Dox has reduced cardiotoxicity and myeloablation side effects in treating T‐ALL mice. Mechanistically, MRIAN degrades into l‐phenylalanine, which inhibits PKM2 activity and reduces ROS levels in MDSCs to disturb their immunosuppressive function and increase their differentiation toward normal myeloid cells. Overall, a novel amino acid metabolite nanomedicine is invented to treat T‐ALL through the combination of leukemic cell targeting and immunosurveillance stimulation.

## Graphical abstract

It is shown Metabolic Reprogramming Immunosurveillance Activation Nanomedicine (MRIAN) targets T‐cell acute lymphoblastic leukemia (T‐ALL) cells and myeloid‐derived suppressor cells (MDSCs) in the bone marrow (BM). MRIAN degrades into L‐Phe to reprogram the energy metabolism of MDSCs, which drives their differentiation toward normal myeloid cells and unarms their immunosuppressive function to reinforce immune surveillance in T‐ALL.

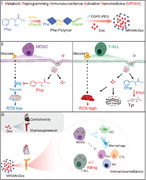

## 1 Introduction

T‐cell acute lymphoblastic leukemia (T‐ALL) is an aggressive hematological cancer from malignant transformed T‐cell progenitors, which affect both children and adults.^[^ ^1^, ^2^ ^]^ Compared with B precursor ALL and myeloid leukemia, T‐ALL patients often suffered a high relapse rate, secondary chemotherapy resistance, and poor prognosis despite the intensified chemotherapy treatment.^[^ ^3^, ^4^ ^]^ Approximately 20% of pediatric and 50% of adult patients with T‐ALL show resistance to the first‐line therapy, fail to obtain a durable complete remission, and die due to disease progression.^[^ ^5^, ^6^, ^7^, ^8^ ^]^

Growing evidence shows that immunosuppressive mechanisms influence the disease initiation and antagonize the desired effects of chemotherapy in hematological malignancies.^[^ ^9^ ^]^ Recent works show that chemoresistance and relapse in leukemia are linked to the compromised immunosurveillance in bone marrow (BM).^[^ ^10^, ^11^, ^12^, ^13^, ^14^ ^]^ Myeloid‐derived suppressor cells (MDSCs) are a heterogeneous population of immature myeloid cells, which expand during cancer and have a remarkable ability to suppress T cells for antitumor responses.^[^ ^15^, ^16^ ^]^ Recent work shows that T‐ALL patients have increased MDSCs in BM, which hinders the immunosurveillance in T‐ALL.^[^ ^17^ ^]^ Therefore, eliminating immunosuppressive MDSCs to reinstate the immunosurveillance offers new opportunities to treat hematological malignancies and prevent relapse.

**Scheme 1:**
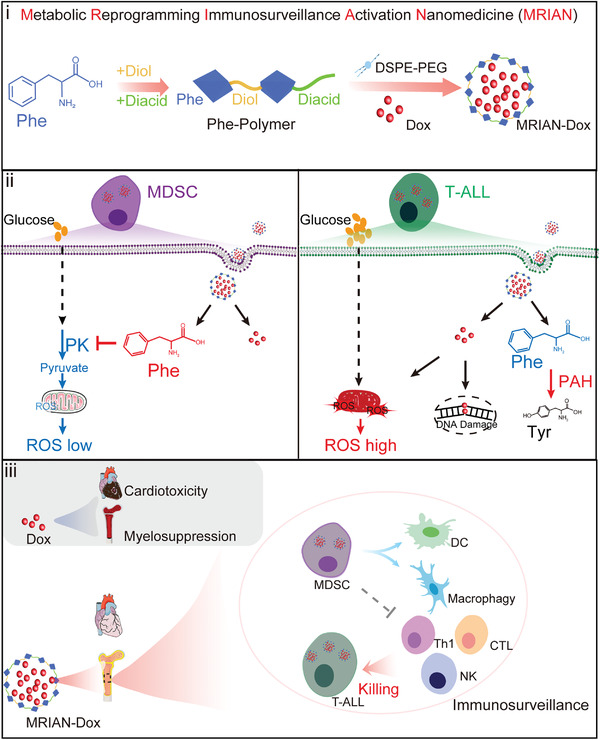
Graphical abstract to illustrate the synthesis process and functional features of Metabolic Reprogramming Immunosurveillance Activation Nanomedicine (MRIAN). MRIAN targets T‐cell acute lymphoblastic leukemia (T‐ALL) cells and myeloid‐derived suppressor cells (MDSCs) in the bone marrow (BM). MRIAN degrades into l‐Phe to reprogram the energy metabolism of MDSCs, which drives their differentiation toward normal myeloid cells and unarms their immunosuppressive function to reinforce immune surveillance in T‐ALL.

Amino acid metabolism controls immune responses and cancer,^[^ ^18^, ^19^ ^]^ which offers a therapeutic approach to treat hematologic malignancies.^[^ ^20^ ^]^ However, a druggable amino acid metabolite to enhance immunosurveillance and improve anti‐leukemia efficacy remains unexplored. Here, through a functional screening of amino acid‐based materials, we developed a l‐phenylalanine‐based Metabolic Reprogramming Immunosurveillance Activation Nanomedicine (MRIAN) to impair the immunosuppressive function of MSDCs in the BM. More importantly, MRIAN loaded Doxorubicin (Dox), MRIAN‐Dox, specifically targeted leukemic cells in murine T‐ALL model but spared normal hematopoietic cells to robustly enhance the antitumor efficacy and limit the myeloablation and cardiotoxicity side‐effects of Dox treatment (**Scheme** **1**). Overall, we invented a nanomedicine from the phenylalanine metabolite approach to activate immune surveillance by eliminating MDSCs and specifically targeting leukemic cells for T‐ALL treatment.

## 2 Result

### 2.1 MRIAN Efficiently Penetrates Bone Marrow and Explicitly Targets Leukemic Cells and MDSCs in T‐ALL Mice

**Figure 1:**
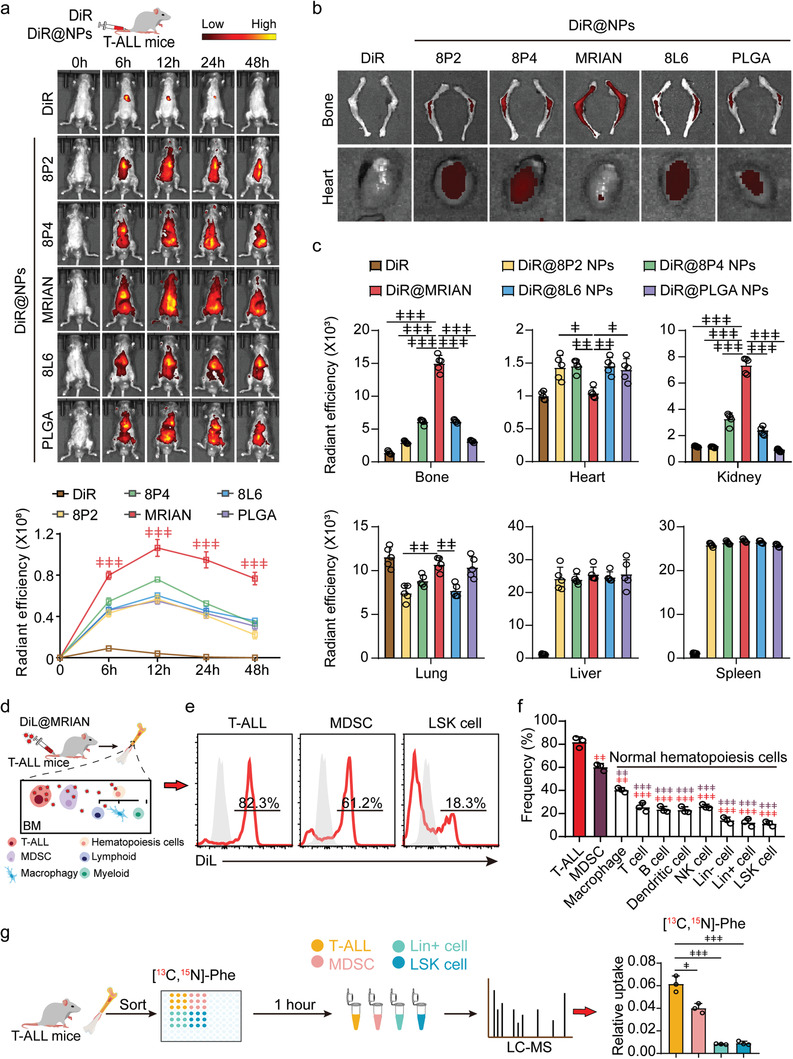
MRIAN efficiently penetrates bone marrow and explicitly targets leukemic cells and MDSCs in T‐ALL mice. a) Bioluminescence images (upper) and quantification (below) of DiR dye intensity in mice treated with DiR dye, DiR@8P2 NPs, DiR@8P4 NPs, DiR@MRIAN, DiR@8L6 NPs, and DiR@PLGA NPs at the indicated time after injection (*n* = 5 mice). b,c) Representative images (b) and quantification (c) of DiR intensity in the indicated organs at 48 h after injection (*n* = 5 mice). d) Scheme for quantifying DiL@MRIAN uptake efficiency by leukemia cells and BM cells in T‐ALL mice. e,f) Representative FACS histogram (e) and quantification (f) of the fluorescent intensity of DiL@MRIAN in T‐ALL cells, MDSCs, and normal hematopoietic cells as indicated (*n* = 5 mice). g) Scheme for stable‐isotope tracer experiment and quantification of cellular uptake of [^13^C, ^15^N] l‐Phe in T‐ALL cells, MDSCs, and normal hematopoietic cells from T‐ALL mice (*n* = 4 mice). Liquid chromatography mass spectrometry (LC–MS). Data represent mean ± s.d. Repeated measures one‐way analysis of variance (ANOVA) followed by Dunnett's test for multiple comparisons, ^ǂ^ *p* < 0.05, ^ǂǂ^ *p* < 0.01, ^ǂǂǂ^ *p* < 0.001.

Leukemic cells reside in the BM niche, which protects them from traditional chemotherapy.^[^ ^21^ ^]^ We used an activated Notch1 mutant driven T‐ALL murine model, which presents over 60% human T‐ALL both phenotypically and genetically,^[^ ^22^, ^23^, ^24^, ^25^ ^]^ to explore a functional nanocarrier that could target leukemic cells in BM (Figure S1a, Supporting Information). Intriguingly, a serial of l‐phenylalanine‐based poly (ester amide) (Phe‐PEA) polymers, especially MRIAN, showed specificity‐enhanced BM accumulation in T‐ALL mice (**Figure** **1**a,b). At 48 h after infusion, MRIAN had ≈3‐fold higher concentration in BM compared to other l‐Phe‐based PEA carriers (8P2 and 8P4) or Leucine‐based PEA carrier (8L6) (Figure 1c). Notably, the BM concentration of MRIAN was ≈4‐fold higher than PLGA nanocarrier, a Food and Drug Administration (FDA) approved polymeric nanocarrier.^[^ ^26^ ^]^ Furthermore, MRIAN was highly accumulated in the liver and spleen, where T‐ALL cells were enriched (Figure S1b, Supporting Information). Conversely, low‐level MRIAN was observed in the heart, indicating the reduced cardiotoxicity potential as a drug carrier (Figure 1b,c); and in the kidney, indicating the high renal metabolic rate of MRIAN (Figure S1b, Supporting Information). Furthermore, we observed that MRIAN was efficiently delivered to the targeted organs as early as 6 h, which was much more efficient than other carriers (Figure 1a). Furthermore, the in vivo concentration of MRIAN was maintained till 48 h, which was higher than other carriers (Figure 1a). Overall, these indicated that MRIAN was an efficient drug carrier for T‐ALL cells in BM.

We further explored the cellular targeting specificity of MRIAN within BM in T‐ALL mice 12 h after infusion (Figure 1d). MRIAN targeted more than 80% of T‐ALL cells, but only about 25–30% of normal hematopoietic cells, including T cells, B cells, dendritic cells (DCs), and natural killer (NK) cells (Figure 1e,f). More importantly, fewer hematopoietic stem and progenitor cells (HSPCs, Lineage^–^Sca1^+^ckit^+^) were targeted by MRIAN (≈18%) (Figure 1e,f). These indicated that MRIAN could precisely deliver drugs into T‐ALL cells but spare normal hematopoietic cells and HSPCs. Intriguingly, we noticed that MRIAN more efficiently targeted MDSCs (60%) than macrophages (40%) in BM (Figure 1e,f). To explore the mechanism, we performed a stable‐isotope tracer metabolic flux experiment to assess the cellular uptake of l‐Phe in T‐ALL mice. Notably, T‐ALL cells and MDSCs had remarkably higher cellular uptake of l‐Phe than normal mature hematopoietic cells (Lineage^+^) and HSPCs (Figure 1g). Moreover, we found that MRIAN can target brain infiltrated T‐ALL cells but spare non‐leukemic normal cells in the brain of T‐ALL mice (Figure S1c–f, Supporting Information).

Overall, our data demonstrated that l‐Phe‐polymer‐based MRIAN could efficiently target T‐ALL cells and MDSCs in T‐ALL mice.

### 2.2 MRIAN‐Dox has Reduced Cardiotoxicity and Tissue Damage than Dox

**Figure 2:**
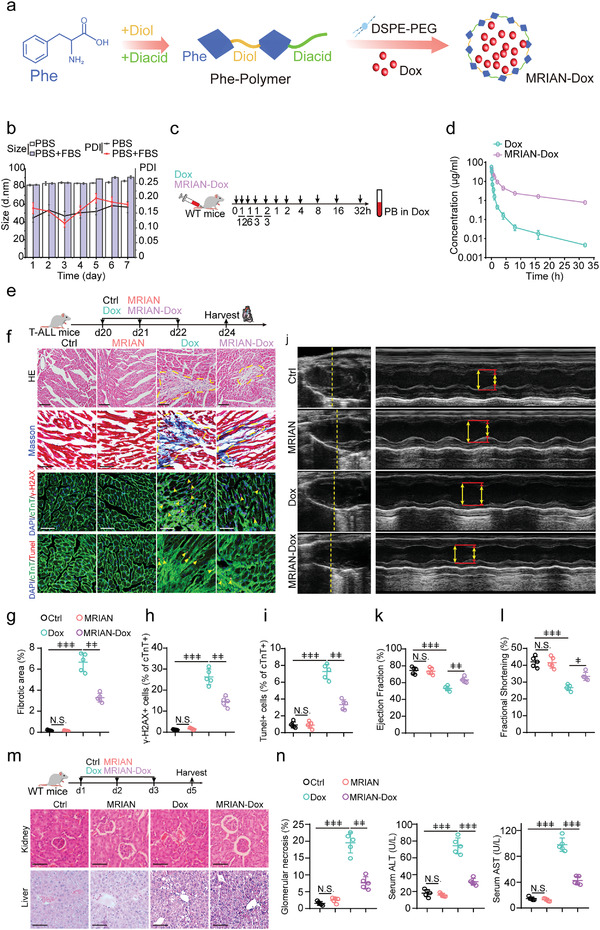
MRIAN‐Dox has reduced cardiotoxicity and tissue damage than Dox. a) Scheme for synthesizing of MRIAN‐Dox. b) Time‐dependent stability of MRIAN‐Dox in PBS or PBS with 10% fetal bovine serum (FBS). c,d) Scheme (c) and pharmacokinetics of Dox and MRIAN‐Dox in mice (d). e) Treatment scheme for T‐cell acute lymphoblastic leukemia (T‐ALL) mice. f–i) HE, Masson staining, representative immunohistochemistry images of TUNEL, *γ*‐H_2_AX (f), quantification of the fibrostic area (g), *γ*‐H_2_AX+ cells frequency (h), and Tunel^+^ cells frequency (i) in the heart section of hearts from T‐ALL mice after indicated treatments (*n* = 5 mice). j–l) M‐mode echocardiography (j), quantification of ejection fraction (k), and fractional shortening (l) of T‐ALL mice after indicated treatments (*n* = 5 mice). m,n) HE of kidney or liver (m) and quantification of glomerular necrosis frequency in the kidney (left) and liver function ALT (middle) and AST (right) in mice serum after indicated treatments. Repeated measures one‐way analysis of variance (ANOVA) followed by Dunnett's test for multiple comparisons, ^ǂ^ *p* < 0.05, ^ǂǂ^ *p* < 0.01, ^ǂǂǂ^ *p* < 0.001.

MRIAN was synthesized via a one‐step polycondensation of dicarboxylic acid‐based monomers and l‐Phe‐based monomers (**Figure** **2**a). The structure of MRIAN was verified by the 1hydrogen‐nuclear magnetic resonance (^1^H‐NMR) (Figure S2a, Supporting Information). Furthermore, MRIAN and Dox were self‐assembled to form spherical nanoparticles with DSPE‐PEG2000 as a stabilizer through a nanoprecipitation method (Figure 2a). To optimize the MRIAN‐Dox with high drug loading and stable properties, we screened the weight ratios of MRIAN to Dox from 2:1 to 9:1 (Figure S2b–d, Supporting Information). Although the particle size and loading capacity (LC) in different weight ratios did not show much difference (Figure S2b,c, Supporting Information), the weight ratio of 9:1 showed the highest encapsulation efficiency (EE) (Figure S2d, Supporting Information). Transmission electron microscopy (TEM) images and size distribution results showed that the MRIAN‐Dox achieved a similar spherical morphology but had slightly decreased particle size than MRIAN (Figure S2e,f, Supporting Information). MRIAN‐Dox maintained their stability in PBS or PBS with 10% fetal bovine serum (FBS) medium for 7 days (Figure 2b). Furthermore, we explored the in vivo pharmacokinetics of MRIAN‐Dox in wild‐type C57BL/6J mice. High‐performance liquid chromatography with fluorescence detection (HPLC‐FLD) showed that the plasma level of MRIAN‐Dox was 100‐fold higher than Dox in mice at 32 h after a single intravenous injection (Figure 2c,d), which indicated that MRIAN‐Dox had a prolonged blood circulation time.

Dox causes dose‐dependent cardiotoxicity by targeting topoisomerase‐IIb to induce DNA double‐strand breaks (DSBs) in cardiomyocytes, which limits its clinical use.^[^ ^27^ ^]^ MRIAN did not accumulate in heart; we therefore assessed the cardiotoxicity of MRAIN‐Dox. We treated T‐ALL mice with Dox or MRIAN‐Dox (3 mg per kilogram of body weight by intravenous injection) every day for 3 days to assess the Dox‐induced cardiomyopathy (Figure 2e). MRIAN‐Dox treated mice had preserved cardiomyocyte cross‐sectional area and reduced collagen deposition compared to mice that received Dox treatment (Figure 2f,g). Furthermore, Dox treatment induced DSBs in cardiomyocytes, which was examined by immunostaining for the DSB marker *γ*‐H2AX,^[^ ^28^ ^]^ leading to cell apoptosis assessed by TUNEL (terminal deoxynucleotidyl transferase dUTP nick end labeling) staining. Notably, the number of DSBs and cell apoptosis caused by MRAIN‐Dox was reduced by 51.7% and 60%, respectively, in cardiomyocytes compared to Dox treatment (Figure 2f,h,i). Furthermore, MRAIN‐Dox induced cardiac troponin T (cTnT) release, an indicator of doxorubicin‐induced cardiotoxicity,^[^ ^29^ ^]^ was dramatically reduced compared to Dox treatment (Figure 2f). Along with the reduction in fibrosis, we observe a partial rescue of both systolic and diastolic cardiac function in MRAIN‐Dox treated mice compared to mice with Dox treatment. Mice receiving MRAIN‐Dox treatment had improved the ejection fraction and shortening fraction compared to mice receiving Dox treatment (Figure 2j–l). Furthermore, our new histology and functional assays showed that MRIAN‐Dox had much lower toxicity on the kidney and liver than Dox treatment in control mice (Figure 2m,n). Overall, our data demonstrated that MRIAN‐Dox treatment had reduced tissue toxicity compared to Dox in mice.

### 2.3 MRIAN has Enhanced Cellular Uptake Efficiently to Induce DNA Damage and Apoptosis in T‐ALL Cells

**Figure 3:**
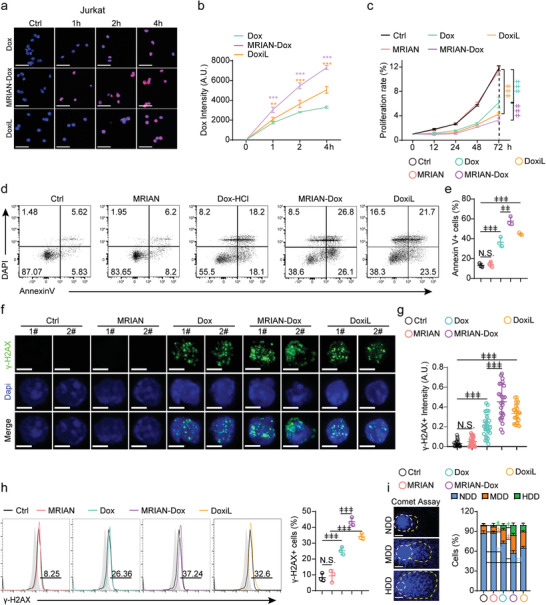
MRIAN‐Dox enhances DNA damage and apoptosis of Jurkat cells induced by Dox in vitro (a,b). Representative images (a) and quantification (b) of cellular uptake of Dox in Jurkat cells at indicated times after incubation with Dox, MRIAN‐Dox, or DoxiL (*n* = 3 independent experiments). c) Cell proliferation rate of Jurkat cells at the indicated time after treatments (*n* = 3 independent experiments). d,e) Representative FACS plots (d) and quantification (e) of Annexin V^+^ apoptotic Jurkat cells at 72 h after treatments (*n* = 3 independent experiments). f,g) Representative image (f) and quantification (g) of *γ*‐H_2_AX staining in Jurkat cells at 72 h after indicated treatments (*n* = 50 cells). h) Representative FACS plots (left) and quantification (right) of *γ*‐H_2_AX positive Jurkat cells at 72 h after indicated treatments (*n* = 3 independent experiments). i) Representative image (left) and quantification (right) of comet assay in Jurkat cells at 72 h after indicated treatments (*n* = 30 cells). Scale bar 50 µm (a) and 5 µm (f,i). Data represent mean ± s.d. Repeated measures one‐way analysis of variance (ANOVA) followed by Dunnett's test for multiple comparisons, ^ǂ^ *p* < 0.05, ^ǂǂ^ *p* < 0.01, ^ǂǂǂ^ *p* < 0.001.

We further investigated the anticancer role of MRIAN‐Dox in T‐ALL cells. MRIAN‐Dox had increased cellular uptake rates than free Dox and liposome‐encapsulated Dox (Doxil)^[^ ^30^ ^]^ in Jurkat cells, a T‐lymphocyte cell line (**Figure** **3**a,b), indicating that MRIAN‐Dox might have enhanced anticancer efficacy for T‐ALL treatment. Indeed, MRIAN‐Dox more robustly inhibited cell proliferation (Figure 3c) and induced apoptosis in Jurkat cells than free Dox and Doxil (Figure 3d,e). Furthermore, we repeatedly observed the enhanced cellular uptake and therapeutic advantages of MRIAN‐Dox than free Dox or Doxil treatment in other two T‐ALL cell lines, DND41 and MOLT4 (Figure S3a–d, Supporting Information).

As Dox induces DNA damage by blocking topoisomerase 2 to trigger DNA damage,^[^ ^31^ ^]^ we further examined the DNA damage rate in T‐ALL cells post‐MRIAN‐Dox treatment. MRIAN‐Dox treatment more efficiently induced DNA damage than free Dox or Doxil treatment in Jurkat cells examined by immunofluorescence staining (Figure 3f,g) and FACS analysis (Figure 3h) for the ratio of *γ*‐H_2_AX positive cells. Furthermore, the MRIAN‐Dox‐induced DNA damage was further confirmed by the single‐cell gel electrophoresis DNA comet assay, in which MRIAN‐Dox induced more moderately damaged DNA (MDD) and heavily damaged DNA (HDD) than free Dox or Doxil treatment (Figure 3i). Furthermore, MRIAN‐Dox more efficiently induced DNA damage in DND41 cells and MOLT4 cells (Figure S3e,f, Supporting Information). However, MRIAN treatment alone did not cause either proliferation inhibition, apoptosis, or DNA damage in Jurkat cells, DND41 cells, or MOLT4 cells (Figure 3c–i and Figure S3c–f, Supporting Information).

Overall, we showed that MRIAN‐Dox had enhanced cellular uptake, which robustly triggered DNA damage and apoptosis of T‐ALL cells in vitro.

### 2.4 MRIAN‐Dox has Enhanced Therapeutic Efficacy to Treat T‐ALL In Vivo

**Figure 4:**
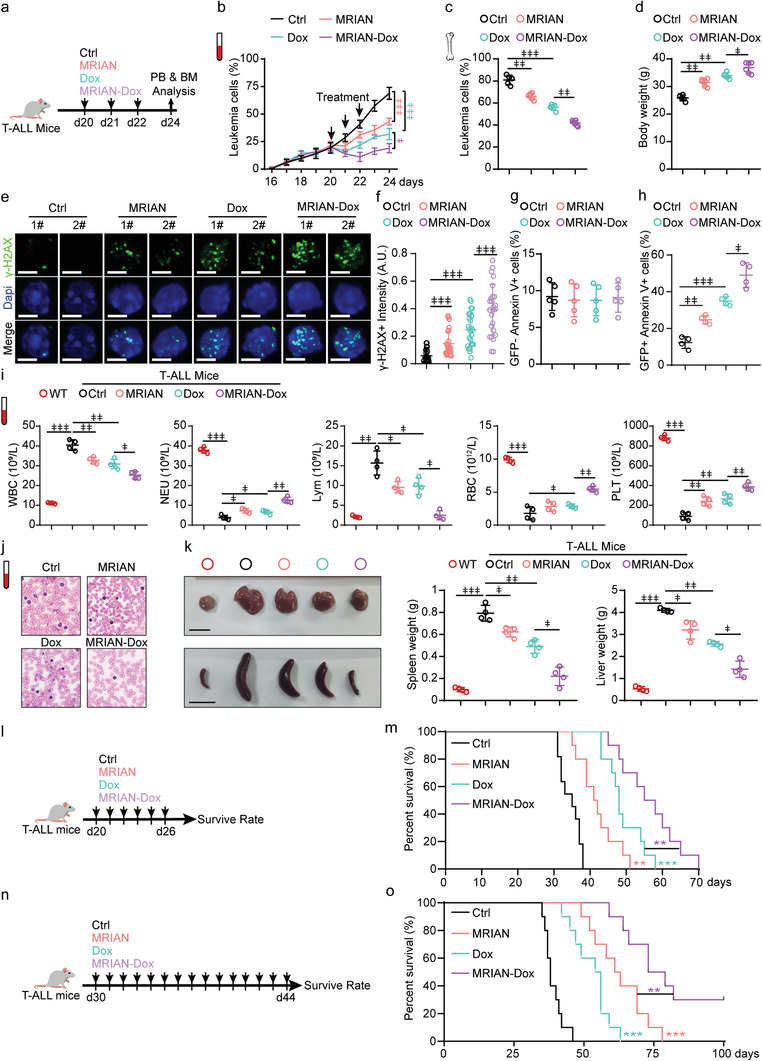
MRIAN‐Dox has enhanced therapeutic efficacy to treat T‐ALL in vivo. a) Treatment scheme for T‐ALL mice. b,c) The frequency of T‐ALL cells in peripheral blood (PB) (b) and bone marrow (BM) (c) at the indicated time after treatments (*n* = 4 mice). Leukemia cells (GFP^+^ cells). d) The body weight of T‐ALL mice 24 days after transplantation with indicated treatment (*n* = 4 mice). e,f) The representative image (e) and quantification (f) of *γ*‐H_2_AX staining in T‐ALL cells from T‐ALL mice 24 days after transplantation with indicated treatment (*n* = 30 cells). Scale bar 5 µm (e). g,h) Quantification of apoptotic cells in GFP^–^ normal hematopoietic cells and GFP^+^ T‐ALL cells from T‐ALL mice 24 days after transplantation with indicated treatment (*n* = 4 mice). i) The blood routine analysis of normal C57B6J without transplantation (WT) and T‐ALL mice 24 days after transplantation with indicated treatment (*n* = 4 mice). j–k) Representative images of the PB smear (j) and the representative images (left) and weight quantification (right) of liver and spleen (k) of T‐ALL mice 24 days after transplantation with indicated treatment (*n* = 4 mice). l,m) Treatment scheme (l) and survival curve T‐ALL mice with indicated treatments (m) (*n* = 10 mice). n,o) Treatment Scheme (n) and survival curve T‐ALL mice with indicated treatments (o) (*n* = 10 mice). Data represent mean ± s.d. Repeated measures one‐way analysis of variance (ANOVA) followed by Dunnett's test for multiple comparisons, ^ǂ^ *p* < 0.05, ^ǂǂ^ *p* < 0.01, ^ǂǂǂ^ *p* < 0.001.

We next investigated the therapeutic efficacy of MRIAN‐Dox in treating T‐ALL in vivo. To this aim, we employed the Notch1‐mutant driven T‐ALL murine model and further applied MRIAN‐Dox treatment with placebo control, Dox treatment, or MRIAN treatment (**Figure** **4**a). Firstly, we observed that MRIAN‐Dox robustly reduced the leukemia burden in peripheral blood (PB), which was significantly more dramatic than the Dox treatment group (*p* = 0.024) (Figure 4b). The enhanced efficacy of MRIAN‐Dox treatment was also observed in BM, in which MRIAN‐Dox treatment reduced more leukemia cells than Dox treatment (Figure 4c). Consequently, the body weight was recovered more efficiently in mice with MRIAN‐Dox treatment than Dox treatment (Figure 4d). Interestingly, we noticed that MRIAN alone also reduced leukemia burden in PB and BM, which was almost as efficient as Dox treatment (Figure 4b,c). Consistently, the MRIAN treatment recovered the bodyweight of T‐ALL mice as efficiently as the Dox treatment (Figure 4d). These observations indicated that MRIAN also had an antitumor effect in vivo.

MRIAN‐Dox treatment induced more DNA damage (Figure 4e,f and Figure S4a–c, Supporting Information), increased higher ROS level (Figure S4d, Supporting Information), and more cellular apoptosis (Figure 4g,h and Figure S4e, Supporting Information) of T‐ALL cells in the BM of mice than Dox treatment. Interestingly, we noticed that MRIAN‐Dox treatment specifically triggered more apoptosis in GFP^+^ T‐ALL cells but not GFP^–^ normal hematopoietic cells compared to Dox treatment (Figure 4g,h, Supporting Information), indicating the reduced myelosuppression side‐effects of MRIAN‐Dox treatment than Dox treatment. Indeed, the MRIAN‐Dox treatment group more efficiently recovered the numbers of neutrophils, red blood cells, and platelets; and reduced leukemic cells in white blood cells and lymphocytes compared to the Dox treatment group (Figure 4i). In line with this, MRIAN‐Dox treatment more efficiently reduced leukemic cells in PB (Figure 4j) and recovered spleen and liver in size and weight (Figure 4k) than Dox treatment in T‐ALL mice. Notably, MRIAN‐Dox treatments for six consecutive days significantly extended the mean overall survival (MOS) to 70 days compared to 38 days in the control group and 58 days in the Dox treatment group (Figure 4l,m). We also noticed that MRIAN treatment alone significantly extended the MOS to 52 days in T‐ALL mice (*p* = 0.0009). As MRIAN‐Dox had reduced toxicity in vivo, we extended the MRIAN‐Dox treatments to 15 consecutive days, initiated at d30 after T‐ALL cell transplantation (Figure 4n). Notably, MRIAN‐Dox treatment extended the MOS to 73 days and saved about 40% of T‐ALL mice surviving more than 100 days (Figure 4n,o). Furthermore, MRIAN treatment alone extended the MOS to 61 days in T‐ALL mice even more efficiently than Dox treatment (MOS 54 days). MRIAN treatment alone did not directly influence T‐ALL cells in vitro but suppressed leukemic cells in vivo, suggesting MRIAN treatment might enhance the antitumor immunosurveillance for T‐ALL treatment.

Overall, our data showed that MRIAN‐Dox treatment had enhanced therapeutic efficacy in vivo than Dox treatment in T‐ALL mice.

### 2.5 MRIAN‐Dox Improves Immune Surveillance in T‐ALL Mice

**Figure 5:**
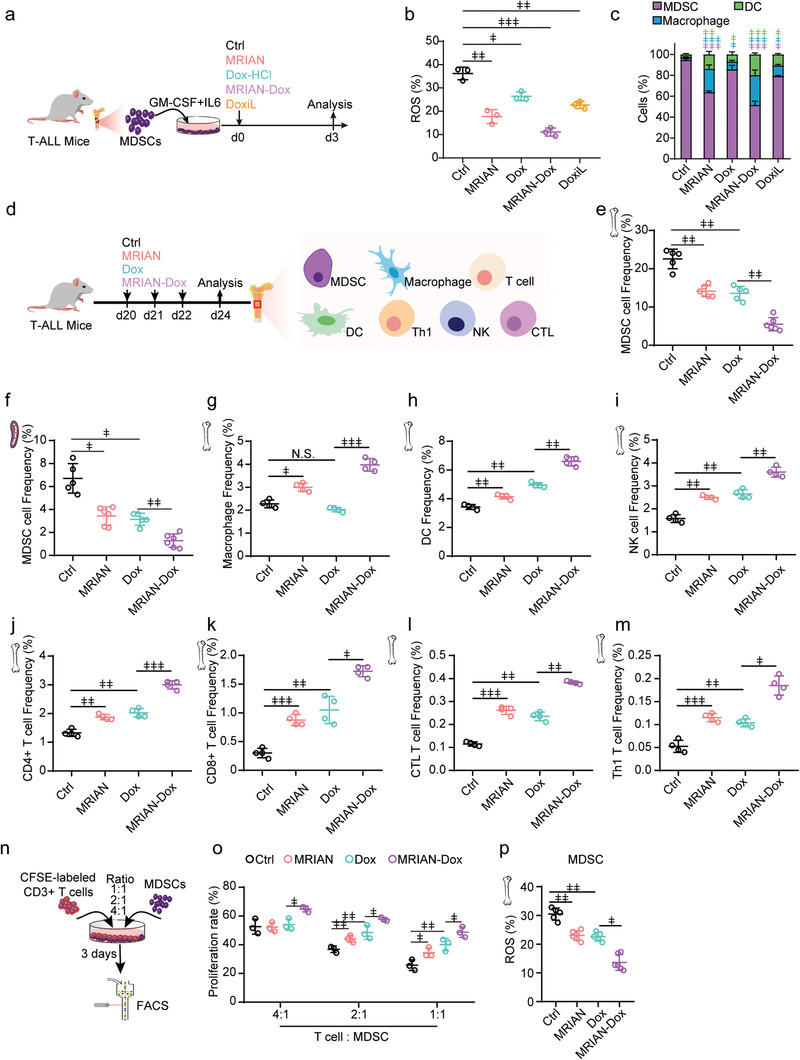
MRIAN‐Dox improved leukemia microenvironment in T‐ALL. a) In vitro MDSC differentiation assay scheme. b,c) ROS level (b) and frequency of MDSCs, macrophages, and dendritic cells (DCs) (c) of purified MDSCs after indicated treatment (*n* = 3 mice). d) Treatment scheme for T‐ALL mice. e,f) The frequency of MDSCs in bone marrow (BM) (e) and spleen (f) in T‐ALL mice after indicated treatments (*n* = 4 mice per group). g–m) The frequency of macrophages (g), DCs (h), NK cells (i), CD4^+^ T cells (j), CD8^+^ T cells (k), CD3^+^CD8^+^IFN*γ* ^+^ cytotoxic T lymphocytes (CTL T cells) (l), and CD3^+^CD4^+^IFN*γ* ^+^ Type 1 T helper cells (Th1 T cells) (m) in BM in T‐ALL mice 24 days after transplantation with indicated treatment (*n* = 4 mice per group). n,o) Experiment scheme (n) and quantification of proliferating T cells after co‐culture with MDSCs with indicated ratios (o). MDSCs were isolated from T‐ALL mice with or without treatment as indicated. p) Quantification of ROS levels in MDSCs from T‐ALL 24 days after transplantation with indicated treatment (*n* = 4 mice per group). Data represent mean ± s.d. Repeated measures one‐way analysis of variance (ANOVA) followed by Dunnett's test for multiple comparisons, ^ǂ^ *p* < 0.05, ^ǂǂ^ *p* < 0.01, ^ǂǂǂ^ *p* < 0.001.

Furthermore, we observed that MRIAN and MRIAN‐Dox efficiently reduced ROS levels and induced differentiation of MDSCs toward macrophages and DCs in vitro (**Figure** **5**a–c). Then, we investigated how MRIAN impacts immune surveillance in T‐ALL mice (Figure 5d and S5a,b, Supporting Information). MRIAN‐Dox treatment reduced the frequency of MDSCs in BM and spleen more efficiently than Dox treatment, which is known to eliminate MDSCs^[^ ^32^ ^]^ (Figure 5e,f). Furthermore, MRIAN also reduced MDSC numbers in BM and spleen as efficiently as Dox treatment (Figure 5e,f). These observations indicated that MARIN and MRIAN‐Dox might facilitate the recovery of immunosurveillance in T‐ALL mice.

We then investigated the numbers of innate and adaptive immune cells in T‐ALL mice after the MRIAN‐Dox treatment. MRIAN‐Dox treatment remarkably recovered the number of macrophages (Figure 5g), DCs (Figure 5h), NKs (Figure 5i), CD4^+^ T cells (Figure 5j), CD8^+^ cells (Figure 5k), CD3^+^CD8^+^IFN*γ* ^+^ cytotoxic T lymphocyte (CTL) (Figure 5l), and CD3^+^CD4^+^IFN*γ* ^+^ Type 1 T helper (Th1 cells) (Figure 5m) more efficiently than Dox treatment in the BM of T‐ALL mice. We also found that MRIAN treatment significantly recovered the number of immune cells as efficiently as Dox treatment (Figure 5e–m). To investigate whether MRIAN‐Dox regulates the immunosuppressive function of MDSCs, we performed a T cell and MDSC co‐culture experiment (Figure 5n). We found that purified MDSCs from MRIAN‐Dox treated T‐ALL mice showed much less efficiency in suppressing T‐cell proliferation than MDSCs from control or Dox treated T‐ALL mice (Figure 5o). Consistently, MRIAN treatment also reduced the immunosuppressive function of MDSCs as efficiently as Dox treatment (Figure 5o).

Furthermore, MRIAN‐Dox and MRIAN treatment efficiently reduced the ROS level in MDSCs from T‐ALL mice (Figure 5p). Consistent with a previous report,^[^ ^32^ ^]^ we also found that Dox treatment reduced the ROS level in MDSCs (Figure 5p). Low ROS levels are known to induce MDSC differentiation toward normal macrophages, DCs, and NK cells.^[^ ^33^ ^]^ Our data showed that MRIAN reduced the ROS level in MDSCs, which enhanced their differentiation to reduce their number and impair their immunosuppressive function to recover the immune surveillance in T‐ALL mice.

### 2.6 MRIAN Inhibits Mitochondrial Metabolism and Reduces ROS Levels in MDSCs

**Figure 6:**
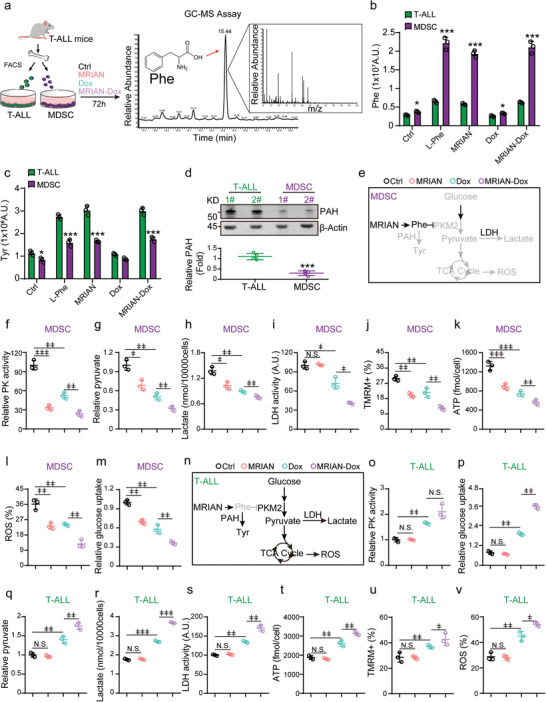
MRIAN reduces MDSC without affecting the metabolic level of leukemia cells. a–c) Scheme and representative gas chromatography mass spectrometry (GC‐MS) spectrum of Phe (a) and quantification (b) for the cellular Phe levels (b) and Tyr levels (c) in T‐ALL cells and MDSCs by GC‐MS after indicated treatments (*n* = 3 replicates per group). d) Western blot analyses (upper) and quantification (down) of PAH protein levels in T‐ALL cells and MDSCs from T‐ALL mice (*n* = 4 mice). 1#, 2# indicated two representative individual T‐ALL mice. e) Scheme of glucose metabolism in MDSCs. f–m) Relative intracellular pyruvate kinase (f), relative intracellular pyruvate concentration (g), lactate production (h), LDH activity (i), mitochondrial activity (TMRM) (j), intracellular ATP concentration (k), Cell ROXDeep (ROS^high^) cell frequency (l), and relative glucose uptake (m) in MDSCs 72 h after indicated treatments (*n* = 4 mice per group). n) Scheme of glucose metabolism in T‐ALL cells. o–v) Relative intracellular pyruvate kinase (o), relative glucose uptake (p), relative intracellular pyruvate concentration (q), lactate production (r), LDH activity (s), intracellular ATP concentration (t), mitochondrial activity (TMRM) (u), and Cell ROXDeep (ROS^high^) cell frequency (v) in T‐ALL cells 72 h after indicated treatments (*n* = 4 mice per group). Data represent mean ± s.d. Two‐tailed Student's *t*‐tests were used to assess statistical significance. *p < 0.05, **p < 0.01, ***p < 0.001. Repeated measures one‐way analysis of variance (ANOVA) followed by Dunnett's test for multiple comparisons, ^ǂ^ *p* < 0.05, ^ǂǂ^ *p* < 0.01, ^ǂǂǂ^ *p* < 0.001.

We further explored the mechanism that MRIAN regulates ROS levels in MDSCs. Mass spectrometry (MS) analysis showed that MRIAN and MRIAN‐Dox specifically increased the l‐Phe level in MDSCs but not in T‐ALL cells (**Figure** **6**a,b). Furthermore, we observed that MRIAN and MRIAN‐Dox specifically increased the tyrosine (Tyr) level in T‐ALL cells (Figure 6c). This was potentially due to the high expression level of phenylalanine hydroxylase (PAH), which catalyzes the hydroxylation of the l‐Phe to generate Tyr^[^ ^34^, ^35^ ^]^ in T‐ALL cells (Figure 6d).

l‐Phe is known to inhibit the activation of pyruvate kinase M2 (PKM2),^[^ ^36^, ^37^, ^38^ ^]^ which catalyzes the final and an irreversible step of glycolysis to generate pyruvate and ATP.^[^ ^39^ ^]^ Therefore, we investigated how MRIAN regulates PKM2 activity and glucose metabolism in MDSCs (Figure 6e). Notably, MRIAN and MRIAN‐Dox treatment dramatically reduced the PKM2 activity in MDSCs (Figure 6f), indicating that MRIAN might reprogram the metabolism profile of MDSCs. Therefore, we further explored the carbohydrate metabolic profile in MDSCs after treatments. As a direct substrate of PKM2, the pyruvate level in MDSCs was dramatically reduced after MRIAN or MRIAN‐Dox treatment (Figure 6g). Furthermore, the reduced pyruvate level in MDSCs resulted in the reduced lactate level (Figure 6h) and LDH activity (Figure 6i), which indicated decreased glycolysis levels in MDSCs after MRIAN or MRIAN‐Dox treatment. Furthermore, we also observed a decreased mitochondrial oxidative phosphorylation level in MDSCs, evidenced by the reduced mitochondrial potential (Figure 6j), decreased ATP production (Figure 6k), and ROS level (Figure 6l) in MSDCs after MRIAN or MRIAN‐Dox treatment. Consequently, the glucose uptake was dramatically reduced in MDSCs after MRIAN or MRIAN‐Dox treatment (Figure 6m). Consistent with a previous report, we also observed that Dox administration decreases ROS production in MDSCs,^[^ ^32^ ^]^ potentially due to the reduced glucose metabolism (Figure 6f–m).

**Figure 7:**
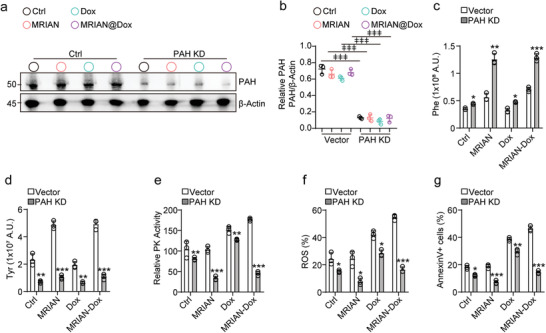
PAH catalyzes Phe and regulates PKM2 activity in T‐ALL cells. a,b) Western blot analyses (a) and quantification (b) of PAH protein levels in Jurkat cells after PAH knockdown with indicated treatment (*n* = 3 independent experiments). c,d) Quantification of gas chromatography mass spectrometry for the cellular Phe levels (c) and Tyr levels (d) in Jurkat cells with indicated treatment (*n* = 3 independent experiments). e–g) Relative intracellular pyruvate kinase (e), ROS level (f), and apoptotic cells (g) in Jurkat cells with indicated treatment (*n* = 3 independent experiments). Data represent mean ± s.d. Two‐tailed Student's *t*‐tests were used to assess statistical significance. *p < 0.05, **p < 0.01, ***p < 0.001. Repeated measures one‐way analysis of variance (ANOVA) followed by Dunnett's test for multiple comparisons, ^ǂ^ *p* < 0.05, ^ǂǂ^ *p* < 0.01, ^ǂǂǂ^ *p* < 0.001.

Furthermore, we investigated how MRIAN regulates glucose metabolism in T‐ALLs (Figure 6n). We found that MRIAN‐Dox and Dox treatment increased glucose metabolism and ROS level in T‐ALL cells (Figure 6o–v). MRIAN‐Dox treatment enhanced PKM2 activity (Figure 6o), glucose uptake (Figure 6p), pyruvate level (Figure 6q), lactate level (Figure 6r), LDH activity (Figure 6s), ATP production (Figure 6t), and mitochondrial potential (Figure 6u) in T‐ALL cells compared to Dox treatment. Consequently, T‐ALL cells have increased ROS levels under MRIAN‐Dox or Dox treatment (Figure 6v). MRIAN‐Dox more efficiently increased the ROS level in T‐ALL cells than Dox treatment (Figure 6v), potentially due to the enhanced cellular drug uptake of MRIAN‐Dox (Figure 3a,b). However, MRIAN did not impact the glucose metabolism in T‐ALL cells (Figure 6o–v). These observations suggested that high PAH activity in T‐ALL cells might compromise the metabolic regulation effect of MARIN by catalyzing the hydroxylation of Phe to generate Tyr. To investigate the role of PAH in T‐ALL cells, we silenced PAH in Jurkat cells (**Figure** **7**a,b). Notably, we found that MRIAN and MRIAN‐Dox efficiently increased the Phe levels but not Tyr levels in PAH‐silenced Jurkat cells (Figure 7c,d). Furthermore, MRIAN and MRIAN‐Dox inhibited the PKM2 activity and reduced ROS levels in PAH‐silenced Jurkat cells (Figure 7e,f). Consequently, the reduced ROS levels attenuated the apoptosis‐induced effects of MRIAN‐Dox in PAH‐silenced Jurkat cells (Figure 7g).

Overall, we demonstrated that MRIAN reprogramed the glucose metabolism and reduced ROS levels in MDSCs, which induced their differentiation and inhibited their immunosuppressive function. Conversely, MRIAN‐Dox treatment caused increased ROS levels and apoptosis in T‐ALL cells due to enhanced cellular uptake and the high PAH activity in T‐ALL cells.

## 3 Discussion

Pyruvate kinase, PKM2, catalyzes the final and irreversible step of glycolysis to generate pyruvate and ATP.^[^ ^39^ ^]^ Restricted PKM2 activity is critical to central anabolic metabolism for tumor growth and leukemia initiation.^[^ ^40^, ^41^ ^]^ l‐Phe inhibits PKM2 activity and manipulates cellular glucose metabolism.^[^ ^42^ ^]^ However, the therapeutic impact remains uncovered, potentially due to the lack of efficient delivery approaches. Tumor‐derived factors stimulate MDSCs with high‐level ROS to maintain their immunosuppressive function.^[^ ^33^ ^]^ Here, we invented an l‐Phe‐based polymer, MRIAN, which specifically targeted MDSCs and degraded into l‐Phe to inhibit PKM2 activity, suppress glucose metabolism, and reduce the ROS levels in MDSCs. Therefore, MRIAN significantly disarmed the immunosuppressive function and induced MDSC differentiation toward functional immune cells, including macrophages, NK cells, and DCs. As MDSCs inhibit T‐cell proliferation during tumor progress,^[^ ^43^, ^44^ ^]^ our data also showed MRIAN significantly recovered T cell number and function by inhibiting MDSCs to reestablish immune surveillance in T‐ALL mice.

Tumor cells undergo metabolic reprogramming to meet their bioenergetic and biosynthetic demands.^[^ ^45^ ^]^ Mitochondrial metabolism provides key metabolites to support tumor anabolism, and fine‐tuning the level of glycolysis offers a therapeutic strategy for leukemia treatment.^[^ ^46^, ^47^ ^]^ Conversely, T‐ALL leukemia stem cells require low ROS levels for disease initiation and resistance to chemotherapy.^[^ ^48^, ^49^, ^50^, ^51^, ^52^ ^]^ Anticancer agent Dox can damage leukemic cells by raising their cellular ROS level, in addition to the DNA DSB triggered apoptosis.^[^ ^53^, ^54^ ^]^ Therefore, low ROS levels can attenuate the cell‐killing effects of chemotherapeutic drugs. Here, we showed that although leukemic cells have basal Phe levels to keep their survival,^[^ ^55^ ^]^ high‐level PAH activity in T‐ALL cells efficiently reduced the excess l‐Phe levels to maintain a stable PKM2 activity and ROS levels after MRIAN treatment. More importantly, MRIAN‐Dox specifically targeted T‐ALL cells in vivo and efficiently enhanced ROS levels to trigger apoptosis in T‐ALL cells by increased Dox cellular uptake. Furthermore, MRIAN‐Dox spared normal hematopoietic cells and HSPCs, which essentially reduced the myeloablation side effect of Dox. However, whether other amino acids have the potential therapeutic effects in leukemia treatment is warranted in future studies. Dox causes cumulative and dose‐dependent cardiotoxic side‐effects in cancer patients.^[^ ^56^ ^]^ Here, we found that MRIAN‐Dox treatment had reduced cardiotoxicity side‐effects than Dox treatment due to tissue‐specific targeting delivery.

Collectively, this study invented MRIAN as a novel metabolic modifier to inhibit MDSCs and a precise drug delivery approach to treat T‐ALL.

## 4 Experimental Section

### Animals

C57BL/6J mice were purchased from the Laboratory Animal Center of Sun Yat‐sen University. For the T‐ALL murine model, MSCV‐NOTCH1‐^Δ^E‐IRES‐GFP infected mouse pre‐leukemia cells (1 × 10^4^) were transplanted into sublethally irradiated (4.5 Gy) recipients (6–8‐week‐old) by intravenous injection together with 2 × 10^5^ CD45.1 rescue cells. GFP^+^ leukemic cells in BM, PB, spleen, and brain were analyzed by using flow cytometry as indicated. MRIAN, Dox (23214‐92‐8, Huateng pharma), and MRIAN‐Dox were intravenously injected at a dose of 3 mg kg^–1^ as indicated. All animal experiments were performed according to protocols approved by the institutional animal care and use committee.

### Synthesis and Characterization of PEA Polymer and Nanoparticles

The synthesis of PEA polymers were performed as the following steps: (i) preparation of di‐p‐nitrophenylesters of dicarboxylic acid (monomer I); (ii) preparation of toluene‐4‐sulfonic acid salts of bis (Phe or Leu) alkylene diesters (monomer II); (iii) preparation of Phe‐PEA polymers through solution polycondensation. The PEA polymer (8P2, 8P4, 8L6) were denoted as “xPy” or “xLy”, which “x” represents the number of methylene groups in dicarboxylic acid, “P” and “L” represent Phe and Leu respectively, and “y” represents the number of methylene groups in diol. The chemical structure of PEA polymers was determined by 1H nuclear magnetic resonance (^1^H NMR, Avance III, Bruker). Poly (D,l‐lactide‐co‐glycolide) (PLGA) was purchased from Sigma‐Aldrich. MRIAN was prepared by a one‐step nanoprecipitation method with Phe‐PEA polymer and DSPE‐PEG2000.^[^ ^57^ ^]^ The polymer nanoparticles and Dox or dye‐loaded polymer (DiR and DiL) were prepared through a simple nanoprecipitation method. Briefly, the different weight ratios of polymer and Dox or dye were dissolved in DMSO and then dropwise added into an aqueous solution containing DSPE‐PEG 2000 under stirring. The final nanoparticles were obtained by using centrifugal filters. The size and zeta potential of nanoparticles were measured by dynamic light scattering (DLS, Zetasizer Nano‐ZS90, Malvern). The LC and EE of Dox or dye‐loaded polymer NPs were determined by fluorescence spectrophotometry (Agilent Technologies).

### In Vivo Imaging System

For the in vivo biodistribution study, the mice were intravenously injected with DiR dye (MX4005, MKBio), DiR@8P2 NPs, DiR@8P4 NPs, DiR@MRIAN, DiR@8L6 NPs, and DiR@PLGA NPs at a dose of 3 mg kg^–1^. Mice were anesthetized and imaged using the in vivo imaging system (IVIS Lumina XRMS, USA) at the indicated time after injection. After 48 h, the bone and organs were analyzed by Living Image Software. The fluorescence signal was quantified using Living Image Software (IVIS Lumina XRMS, USA).

### Plasma Pharmacokinetics of Dox and MRIAN‐Dox In Vivo

An amount of 3 mg kg^–1^ Dox and MRIAN‐Dox were intravenously injected into C57BL/6J mice as indicated. Dox concentration was determined by measuring fluorescence at 485 nm excitation and 590 nm emission in PB, using the multilabel counter model 1420 VICTOR (PerkinElmer, USA).

### Cell Lines and In Vitro Culture

The human T lymphoblastic cell lines Jurkat (ATCC Number, TIB‐152), MOLT4 (ATCC Number, CRL‐1582) were purchased from ATCC. DND41 cell line was kindly provided by Prof. Hudan Liu (Wuhan University, China). MRIAN, Dox, l‐Phe (P2126, Sigma), DoxiL (CSPC Pharmaceutical Group), and MRIAN‐Dox were treated with 0.5 × 10^−6^ m for indicated. T‐ALL cell lines, which were grown in RPMI 1640 medium supplemented with 10% fetal serum and 1% l‐glutamine (Life Technologies, Grand Island, NY, USA) (complete RPMI).

### Flow Cytometry

Leukemia cells were analyzed from BM (femur and tibia), spleen, brain, or PB as indicated. DiL dye was purchased from Beyotime (C1036). Monoclonal antibodies, anti‐CD3 (145‐211, Biolegend), anti‐CD4 (GK1.5, ebioscience), anti‐CD8 (53‐6.7, ebioscience), anti‐B220 (RA3‐6B2, Biolegend), anti‐IFN*γ* (XMG1.2, ebioscience), anti‐CD11b (M1/70, ebioscience), anti‐IgM (II/41, Biolegend), anti‐CD45 (30‐F11, ebioscience), anti‐TER119 (TER‐119, Biolegend), anti‐Gr1 (RB6‐8C5, ebioscience), anti‐Sca‐1 (D7, ebioscience), anti‐c‐Kit (2B8, ebioscience), anti‐CD11c (2B8, ebioscience), anti‐NK1.1 (PK136, ebioscience), and anti‐F4/80 (BM8, ebioscience) (all used as 50 ng per million cells) were used where indicated. For normal mature hematopoietic cells, a lineage cocktail (Lin, phycoerythrin (PE)‐Cy5) was used, including anti‐CD3, anti‐CD4, anti‐CD8, anti‐CD11b, anti‐Gr1, anti‐B220, anti‐IgM, and anti‐TER119. For DNA damage, the cells were fixed, permeabilized, and stained by anti‐*γ*‐H_2_AX (613 404, Biolegend). The cells were further incubated with 0.1 µg µL^–1^ DAPI (1306, Thermo Scientific) for 30 min at room temperature. For apoptosis, ROS, and mitochondrial activity analysis, the cells were stained by AnnexinV (640 945, Biolegend), or DHE (5 × 10^−6^ m KGAF019, KeyGEN), or Tetramethylrhodamine (TMRM, 20 × 10^−9^ m, T668, Thermo Fisher Scientific). Cell sorting and analysis were performed using an Attune NxT analyzer (Thermo Fisher Scientific) or InFlux Cell Sorter (BD Biosciences). Data were analyzed by using FlowJo software.

### Immunostaining

Jurkat cells, DND41, MOLT4, purified leukemia cells, and tissue sections were fixed by 4% paraformaldehyde (PFA). Anti‐*γ*‐H_2_AX (rabbit, 1:100, 7631S, Cell Signaling Technology) or anti‐cTnT (MA5‐12960, ThermoFisher) were stained, followed by a donkey anti‐rabbit AF488 (1:500, A21206, Thermo Scientific) secondary antibody staining. The apoptotic cardiomyocytes were detected by TUNEL assay according to the manufacturer's instruction (C1088, Beyotime). For high‐resolution three‐dimensional images, z‐stack collected images from Nikon C2plus were analyzed with ImageJ (National Institutes of Health).

### Histology and Function Analysis for Tissues

The hearts, liver, and kidney were fixed by 4% PFA for overnight, embedded in paraffin or OCT, and sectioned at 7 µm. Slides were stained with Masson's trichrome (ab150686, Abcam) and HE (C0105S, Beyotime) to identify areas of fibrosis and pathology. The levels of aspartate aminotransferase (AST, C010‐2, Nanjing jiancheng) and Alanine aminotranferease (ALT, C009‐2, Nanjing jiancheng) in serum were measured according to the manufacturer's instructions.

### Echocardiography

Cardiac function was determined by echocardiography according to the manufacturer's instructions (VisualSonics, Vevo 2100, 40MHz 550S probe).

### Comet Assay

The alkaline comet assay was performed according to the manufacturer's instruction (Trevigen, 4250‐050‐K).

### Western Blotting

The equal number of cells were purified by FACS and harvested in PBS with 2% FBS. The cells were then washed by PBS and lysed by RIPA. Equal amounts of protein extracts were fractionated by 12.5% SDS‐PAGE and transferred to a PVDF membrane (IPVH00010, Merck Millipore). After blocked with 5% non‐fat milk in Tris‐buffered saline with Tween‐20 (TBST, pH 7.6) for 1 h at room temperature, the membranes were incubated with primary antibodies, anti‐PAH (rabbit, 1:1000, 16347‐1‐AP, Proteintech) and anti‐*β*‐Actin (rabbit, 1:1000, 4970s, Cell Signaling Technology) overnight at 4 °C and then incubated with secondary antibodies (rabbit, 1:10000, W401B, Promega) for 1 h at room temperature. Western blot signals were detected by digital imaging with a charge‐coupled device camera system (Odyssey Fc). The images shown are representative of images from at least three experiments.

### Glucose Uptake

For measurement of glucose uptake, 2 × 10^4^ leukemia cells and MDSC were cultured in 200 µL of glucose, glutamine, pyruvate free medium containing 100 × 10^−6^ m of 2‐(n‐(7‐nitrobenz‐2‐oxa‐1,3‐diazol‐4‐ylamino)‐2‐deoxyglucose (2‐NBD‐Glucose, 2NBDG, 11 046, Cayman Chemical) for 2 h after which cells were then detected fluoresces (excitation/emission = 485/650 nm) by the Microplate reader (Spectra Max).

### Metabolic State Analysis

Sorted leukemia cells and MDSCs (1 × 10^5^) were lysed, and intracellular ATP, LDH activity, pyruvate content, and NAD^+^/NADH ratio were measured using the ATP Determination Kit (S0026, Beyotime), the LDH Cytotoxicity Detection Kit (C0017, Beyotime), the Pyruvate Assay Kit (BC2205, Solarbio), and the NAD^+^/NADH ratio Assay Kit (S0175, Beyotime) respectively, according to the manufacturers' instructions. For Lactate production, sorted leukemia cells and MDSCs (1 × 10^5^) were incubated in StemSpan serum‐free medium (Stem Cell Technologies, 9650) for 18 h at 37 °C. Supernatant cells were then analyzed using the Lactate Assay Kit (BC2235, Solarbio) according to the manufacturer's instructions.

### Blood Routine Examination

All blood samples underwent blood routine examination using a ProCyte Dx Hematology Analyzer (IDEXX).

### T‐cell Suppression Assay

MDSCs were FACS purified from T‐ALL leukemia BM. CD3^+^ T cells were FACS purified from the spleen of wild‐type C57BL/6J mice. CD3^+^ T cells were further labeled by CFSE (Invitrogen) and cultured in 96‐well plates coated with anti‐CD3 (MAB4841, R&D) and anti‐CD28 (MAB4832, R&D) antibodies. MDSCs and T cells were cultured with ratios of 1:1, 1:2, and 1:4.

### Virus Infection

5 × 10^6^ Jurkat cells were infected with PAH shRNA (GCTGGACAGATTTGCCAATCA) or control lentivirus‐shRNA (random sequence) in Psico‐EF*α*‐IRES2‐EGFP vector. Cells were cultured for 16 h before incubating with virus media for 8 h and cultured in a fresh medium for 3 days for subsequent analysis.

### In Vitro MDSCs Differentiation Assay

A total number of 5 × 10^5^ MDSCs were FACS purified from T‐ALL leukemia BM and cultured in 24‐well plates with a complete culture medium supplemented with IL‐6 (10 ng mL^–1^; R&D Systems) and GM‐CSF (10 ng mL^–1^; R&D Systems).

### Phe and Tyr Quantitation by Mass Spectrometry

A total number of 1 × 10^6^ leukemia cells and MDSC were isolated by FACS. Purified cells were subsequently extracted and analyzed on gas chromatography‐MS (GC‐MS, Thermo, Q Exactive GC). The data were performed feature extraction and preprocessed with Xcalibur 4.3 software (Thermo), and then normalized and edited into a two‐dimensional data matrix by excel 2010 software, including retention time (RT), compound molecular weight (compMW), observations (samples), and peak intensity.

### Metabolic Flux

A total number of 5 × 10^5^ leukemia cells, MDSCs, Lineage^+^ cells, and LSK cells were isolated by flow cytometry and incubated with stable isotope substrate uniformly ^13^C,^15^N‐labeled amino acids (Cambridge Isotope Laboratories, MSK‐A2‐US‐1.2) for 1 h. Cells were subsequently extracted and analyzed on LC‐MS (Agilent, 6495C LC/MS). We calculated the labeled isotopologues as a percentage of the total (labeled and unlabeled amino acids), normalized to the relative abundance of each substrate.

### Statistical Analyses

Data are expressed as mean ± s.d. Experiments were analyzed by Student's *t*‐test *p < 0.05, **p < 0.01, ***p < 0.001; or repeated measures one‐way analysis of variance (ANOVA) followed by Dunnett's test for multiple comparisons ^ǂ^ *p* < 0.05, ^ǂǂ^ *p* < 0.01, ^ǂǂǂ^ *p* < 0.001 as indicated. The survival of the two groups was analyzed using a log‐rank test. Differences were considered statistically significant if *p* < 0.05.

## Conflict of Interest

The authors declare no conflict of interest.

## Author Contributions

C.L., X.Y., and X.X contributed equally to this work. C.L., X.Y., and X.X. designed and performed most of the experiments and analyzed the data. B.W., Y.L., C.J., Y.L., Z.Y., X.T., Y.W., and Z.H. contributed to animal experiments and transcriptional assay. X.Y., T.T., and C.D. contributed to nanocarriers. J.W. and L.J. contributed to the discussion. W.J. and M.Z. wrote the paper and supervised the project.

## Supporting information

Supporting Information

## Data Availability

The data that support the findings of this study are available on request from the corresponding author. The data are not publicly available due to privacy or ethical restrictions.

## References

[1] L. Belver , A. Ferrando , Nat. Rev. Cancer 2016, 16, 494. 27451956 10.1038/nrc.2016.63

[2] C.‐H. Pui , L. L. Robison , A. T. Look , Lancet 2008, 371, 1030. 18358930 10.1016/S0140-6736(08)60457-2

[3] C. H. Pui , W. E. Evans , Semin. Hematol. 2013, 50, 185. 23953334 10.1053/j.seminhematol.2013.06.007 PMC3771494

[4] D. Bhojwani , C. H. Pui , Lancet Oncol. 2013, 14, e205. 23639321 10.1016/S1470-2045(12)70580-6

[5] D. I. Marks , E. M. Paietta , A. V. Moorman , S. M. Richards , G. Buck , G. DeWald , A. Ferrando , A. K. Fielding , A. H. Goldstone , R. P. Ketterling , M. R. Litzow , S. M. Luger , A. K. McMillan , M. R. Mansour , J. M. Rowe , M. S. Tallman , H. M. Lazarus , Blood 2009, 114, 5136. 19828704 10.1182/blood-2009-08-231217 PMC2792210

[6] J. M. Goldberg , L. B. Silverman , D. E. Levy , V. K. Dalton , R. D. Gelber , L. Lehmann , H. J. Cohen , S. E. Sallan , B. L. Asselin , J. Clin. Oncol. 2003, 21, 3616. 14512392 10.1200/JCO.2003.10.116

[7] R. H. Ko , L. Ji , P. Barnette , B. Bostrom , R. Hutchinson , E. Raetz , N. L. Seibel , C. J. Twist , E. Eckroth , R. Sposto , J. Clin. Oncol. 2010, 28, 648. 19841326 10.1200/JCO.2009.22.2950 PMC2815999

[8] A. H. Goldstone , S. M. Richards , H. M. Lazarus , M. S. Tallman , G. Buck , A. K. Fielding , A. K. Burnett , R. Chopra , P. H. Wiernik , L. Foroni , E. Paietta , M. R. Litzow , D. I. Marks , J. Durrant , A. McMillan , I. M. Franklin , S. Luger , N. Ciobanu , J. M. Rowe , Blood 2008, 111, 1827. 18048644 10.1182/blood-2007-10-116582

[9] M. H. Andersen , Leukemia 2014, 28, 1784. 24691076 10.1038/leu.2014.108

[10] E. Vacchelli , Y. Ma , E. E. Baracco , A. Sistigu , D. P. Enot , F. Pietrocola , H. Yang , S. Adjemian , K. Chaba , M. Semeraro , M. Signore , A. De Ninno , V. Lucarini , F. Peschiaroli , L. Businaro , A. Gerardino , G. Manic , T. Ulas , P. Günther , J. L. Schultze , O. Kepp , G. Stoll , C. Lefebvre , C. Mulot , F. Castoldi , S. Rusakiewicz , S. Ladoire , L. Apetoh , J. M. Bravo‐San Pedro , M. Lucattelli , et al., Science 2015, 350, 972. 26516201 10.1126/science.aad0779

[11] L. Zitvogel , L. Galluzzi , M. J. Smyth , G. Kroemer , Immunity 2013, 39, 74. 23890065 10.1016/j.immuni.2013.06.014

[12] A. M. Paczulla , K. Rothfelder , S. Raffel , M. Konantz , J. Steinbacher , H. Wang , C. Tandler , M. Mbarga , T. Schaefer , M. Falcone , E. Nievergall , D. Dörfel , P. Hanns , J. R. Passweg , C. Lutz , J. Schwaller , R. Zeiser , B. R. Blazar , M. A. Caligiuri , S. Dirnhofer , P. Lundberg , L. Kanz , L. Quintanilla‐Martinez , A. Steinle , A. Trumpp , H. R. Salih , C. Lengerke , Nature 2019, 572, 254. 31316209 10.1038/s41586-019-1410-1 PMC6934414

[13] Y. Miao , H. Yang , J. Levorse , S. Yuan , L. Polak , M. Sribour , B. Singh , M. D. Rosenblum , E. Fuchs , Cell 2019, 177, 1172. 31031009 10.1016/j.cell.2019.03.025 PMC6525024

[14] J. M. Perry , F. Tao , A. Roy , T. Lin , X. C. He , S. Chen , X. Lu , J. Nemechek , L. Ruan , X. Yu , D. Dukes , A. Moran , J. Pace , K. Schroeder , M. Zhao , A. Venkatraman , P. Qian , Z. Li , M. Hembree , A. Paulson , Z. He , D. Xu , T. H. Tran , P. Deshmukh , C. T. Nguyen , R. M. Kasi , R. Ryan , M. Broward , S. Ding , E. Guest , K. August , A. S. Gamis , A. Godwin , G. S. Sittampalam , S. J. Weir , L. Li , Nat. Cell Biol. 2020.

[15] D. I. Gabrilovich , S. Nagaraj , Nat. Rev. Immunol. 2009, 9, 162. 19197294 10.1038/nri2506 PMC2828349

[16] J. E. Talmadge , D. I. Gabrilovich , Nat. Rev. Cancer 2013, 13, 739. 24060865 10.1038/nrc3581 PMC4358792

[17] H. Hohtari , O. Brück , S. Blom , R. Turkki , M. Sinisalo , P. E. Kovanen , O. Kallioniemi , T. Pellinen , K. Porkka , S. Mustjoki , Leukemia 2019, 33, 1570. 30635636 10.1038/s41375-018-0360-1 PMC6755974

[18] S. Sivanand , M. G. Vander Heiden , Cancer Cell 2020, 37, 147. 32049045 10.1016/j.ccell.2019.12.011 PMC7082774

[19] B. Kelly , E. L. Pearce , Cell Metab. 2020, 32, 154. 32649859 10.1016/j.cmet.2020.06.010

[20] Y. Tabe , P. L. Lorenzi , M. Konopleva , Blood 2019, 134, 1014. 31416801 10.1182/blood.2019001034 PMC6764269

[21] F. Ishikawa , S. Yoshida , Y. Saito , A. Hijikata , H. Kitamura , S. Tanaka , R. Nakamura , T. Tanaka , H. Tomiyama , N. Saito , M. Fukata , T. Miyamoto , B. Lyons , K. Ohshima , N. Uchida , S. Taniguchi , O. Ohara , K. Akashi , M. Harada , L. D. Shultz , Nat. Biotechnol. 2007, 25, 1315. 17952057 10.1038/nbt1350

[22] A. P. Weng , A. A. Ferrando , W. Lee , J. P. t. Morris , L. B. Silverman , C. Sanchez‐Irizarry , S. C. Blacklow , A. T. Look , J. C. Aster , Science 2004, 306, 269. 15472075 10.1126/science.1102160

[23] W. S. Pear , J. C. Aster , M. L. Scott , R. P. Hasserjian , B. Soffer , J. Sklar , D. Baltimore , J. Exp. Med. 1996, 183, 2283. 8642337 10.1084/jem.183.5.2283 PMC2192581

[24] H. Medyouf , X. Gao , F. Armstrong , S. Gusscott , Q. Liu , A. L. Gedman , L. H. Matherly , K. R. Schultz , F. Pflumio , M. J. You , A. P. Weng , Blood 2010, 115, 1175. 20008304 10.1182/blood-2009-04-214718 PMC2826229

[25] T. Sanda , X. Li , A. Gutierrez , Y. Ahn , D. S. Neuberg , J. O'Neil , P. R. Strack , C. G. Winter , S. S. Winter , R. S. Larson , H. von Boehmer , A. T. Look , Blood 2010, 115, 1735. 20007543 10.1182/blood-2009-07-235143 PMC2832805

[26] C. E. Astete , C. M. Sabliov , J. Biomater. Sci., Polym. Ed. 2006, 17, 247. 16689015 10.1163/156856206775997322

[27] S. Zhang , X. Liu , T. Bawa‐Khalfe , L. S. Lu , Y. L. Lyu , L. F. Liu , E. T. Yeh , Nat. Med. 2012, 18, 1639. 23104132 10.1038/nm.2919

[28] D. Plesca , S. Mazumder , A. Almasan , Methods in Enzymol. 2008, 446, 107. 18603118 10.1016/S0076-6879(08)01606-6 PMC2911482

[29] E. H. Herman , S. E. Lipshultz , N. Rifai , J. Zhang , T. Papoian , Z. X. Yu , K. Takeda , V. J. Ferrans , Cancer Res. 1998, 58, 195. 9443390

[30] Y. Barenholz , J. Controlled Release 2012, 160, 117. 10.1016/j.jconrel.2012.03.020 22484195

[31] K. M. Tewey , T. C. Rowe , L. Yang , B. D. Halligan , L. F. Liu , Science 1984, 226, 466. 6093249 10.1126/science.6093249

[32] D. Alizadeh , M. Trad , N. T. Hanke , C. B. Larmonier , N. Janikashvili , B. Bonnotte , E. Katsanis , N. Larmonier , Cancer Res. 2014, 74, 104. 24197130 10.1158/0008-5472.CAN-13-1545 PMC3896092

[33] K. Ohl , K. Tenbrock , Front. Immunol. 2018, 9, 2499. 30425715 10.3389/fimmu.2018.02499 PMC6218613

[34] P. F. Fitzpatrick , Annu. Rev. Biochem. 1999, 68, 355. 10872454 10.1146/annurev.biochem.68.1.355

[35] S. Kaufman , J. Biol. Chem. 1958, 230, 931. 13525410

[36] A. Miller , R. Hawkins , R. Veech , Science 1973, 179, 904. 4734564 10.1126/science.179.4076.904

[37] D. Y. Gui , C. A. Lewis , M. G. Vander Heiden , Sci. Signaling 2013, 6, pe7. 10.1126/scisignal.2003925 23423437

[38] J. A. Macpherson , A. Theisen , L. Masino , L. Fets , P. C. Driscoll , V. Encheva , A. P. Snijders , S. R. Martin , J. Kleinjung , P. E. Barran , Elife 2019, 8, e45068. 31264961 10.7554/eLife.45068 PMC6636998

[39] H. R. Christofk , M. G. Vander Heiden , N. Wu , J. M. Asara , L. C. Cantley , Nature 2008, 452, 181. 18337815 10.1038/nature06667

[40] Y. ‐ H. Wang , D. Scadden , J. Immunol. 2012, 188, 111.19.22140254

[41] A. E. Allen , J. W. Locasale , Cancer Cell 2018, 33, 337. 29533776 10.1016/j.ccell.2018.02.008 PMC6237085

[42] J. A. Macpherson , A. Theisen , L. Masino , L. Fets , P. C. Driscoll , V. Encheva , A. P. Snijders , S. R. Martin , J. Kleinjung , P. E. Barran , F. Fraternali , D. Anastasiou , eLife 2019, 8, e45068. 31264961 10.7554/eLife.45068 PMC6636998

[43] Y. Nefedova , M. Fishman , S. Sherman , X. Wang , A. A. Beg , D. I. Gabrilovich , Cancer Res. 2007, 67, 11021. 18006848 10.1158/0008-5472.CAN-07-2593

[44] S. Nagaraj , K. Gupta , V. Pisarev , L. Kinarsky , S. Sherman , L. Kang , D. L. Herber , J. Schneck , D. I. Gabrilovich , Nat. Med. 2007, 13, 828. 17603493 10.1038/nm1609 PMC2135607

[45] R. J. DeBerardinis , N. S. Chandel , Sci. Adv. 2016, 2, e1600200. 27386546 10.1126/sciadv.1600200 PMC4928883

[46] K. Vasan , M. Werner , N. S. Chandel , Cell Metab. 2020, 32, 341. 32668195 10.1016/j.cmet.2020.06.019 PMC7483781

[47] Y. H. Wang , W. J. Israelsen , D. Lee , V. W. C. Yu , N. T. Jeanson , C. B. Clish , L. C. Cantley , M. G. Vander Heiden , D. T. Scadden , Cell 2014, 158, 1309. 25215489 10.1016/j.cell.2014.07.048 PMC4197056

[48] V. Giambra , C. R. Jenkins , H. Wang , S. H. Lam , O. O. Shevchuk , O. Nemirovsky , C. Wai , S. Gusscott , M. Y. Chiang , J. C. Aster , R. K. Humphries , C. Eaves , A. P. Weng , Nat. Med. 2012, 18, 1693. 23086478 10.1038/nm.2960 PMC3738873

[49] H. Ye , B. Adane , N. Khan , T. Sullivan , M. Minhajuddin , M. Gasparetto , B. Stevens , S. Pei , M. Balys , J. M. Ashton , D. J. Klemm , C. M. Woolthuis , A. W. Stranahan , C. Y. Park , C. T. Jordan , Cell Stem Cell 2016, 19, 23. 27374788 10.1016/j.stem.2016.06.001 PMC4938766

[50] E. D. Lagadinou , A. Sach , K. Callahan , R. M. Rossi , S. J. Neering , M. Minhajuddin , J. M. Ashton , S. Pei , V. Grose , K. M. O'Dwyer , J. L. Liesveld , P. S. Brookes , M. W. Becker , C. T. Jordan , Cell Stem Cell 2013, 12, 329. 23333149 10.1016/j.stem.2012.12.013 PMC3595363

[51] E. R. Lechman , B. Gentner , S. W. K. Ng , E. M. Schoof , P. van Galen , J. A. Kennedy , S. Nucera , F. Ciceri , K. B. Kaufmann , N. Takayama , S. M. Dobson , A. Trotman‐Grant , G. Krivdova , J. Elzinga , A. Mitchell , B. Nilsson , K. G. Hermans , K. Eppert , R. Marke , R. Isserlin , V. Voisin , G. D. Bader , P. W. Zandstra , T. R. Golub , B. L. Ebert , J. Lu , M. Minden , J. C. Y. Wang , L. Naldini , J. E. Dick , Cancer Cell 2016, 29, 214. 26832662 10.1016/j.ccell.2015.12.011 PMC4749543

[52] K. Naka , T. Hoshii , T. Muraguchi , Y. Tadokoro , T. Ooshio , Y. Kondo , S. Nakao , N. Motoyama , A. Hirao , Nature 2010, 463, 676. 20130650 10.1038/nature08734

[53] R. L. Momparler , M. Karon , S. E. Siegel , F. Avila , Cancer Res. 1976, 36, 2891. 1277199

[54] F. A. Fornari , J. K. Randolph , J. C. Yalowich , M. K. Ritke , D. A. Gewirtz , Mol. Pharmacol. 1994, 45, 649. 8183243

[55] H. L. Wang , H. A. Waisman , J. Lab. Clin. Med. 1961, 57, 73. 13783039

[56] K. Chatterjee , J. Zhang , N. Honbo , J. S. Karliner , Cardiology 2010, 115, 155. 20016174 10.1159/000265166 PMC2848530

[57] J. Wu , C. C. Chu , Acta Biomater. 2012, 8, 4314. 22842040 10.1016/j.actbio.2012.07.027

