# Supplementary material for: A Metabolic Reprogramming Amino Acid Polymer as an Immunosurveillance Activator and Leukemia Targeting Drug Carrier for T‐Cell Acute Lymphoblastic Leukemia
Source: Adv Sci (Weinh). 2022 Jan 26;9(9):2104134. doi: 10.1002/advs.202104134 (PMC8948613; doi:10.1002/advs.202104134)
Supplement: Supplementary file 1 — Supporting Information [file ADVS-9-2104134-s001.pdf]

ADVANCED SCIENCE
Open Access

Supporting Information

for *Adv. Sci.*, DOI: 10.1002/advs.202104134

A metabolic reprogramming amino acid polymer as an immunosurveillance activator and leukemia targeting drug carrier for T-cell acute lymphoblastic leukemia

Changzheng Li, Xinru You, Xi Xu, Binghuo Wu, Yuye Liu, Tong Tong, Jie Chen, Yishan Li, Chunlei Dai, Zhitao Ye, Xiaobin Tian, Yan Wei, Zechen Hao, Linjia Jiang, Jun Wu*, and Meng Zhao**

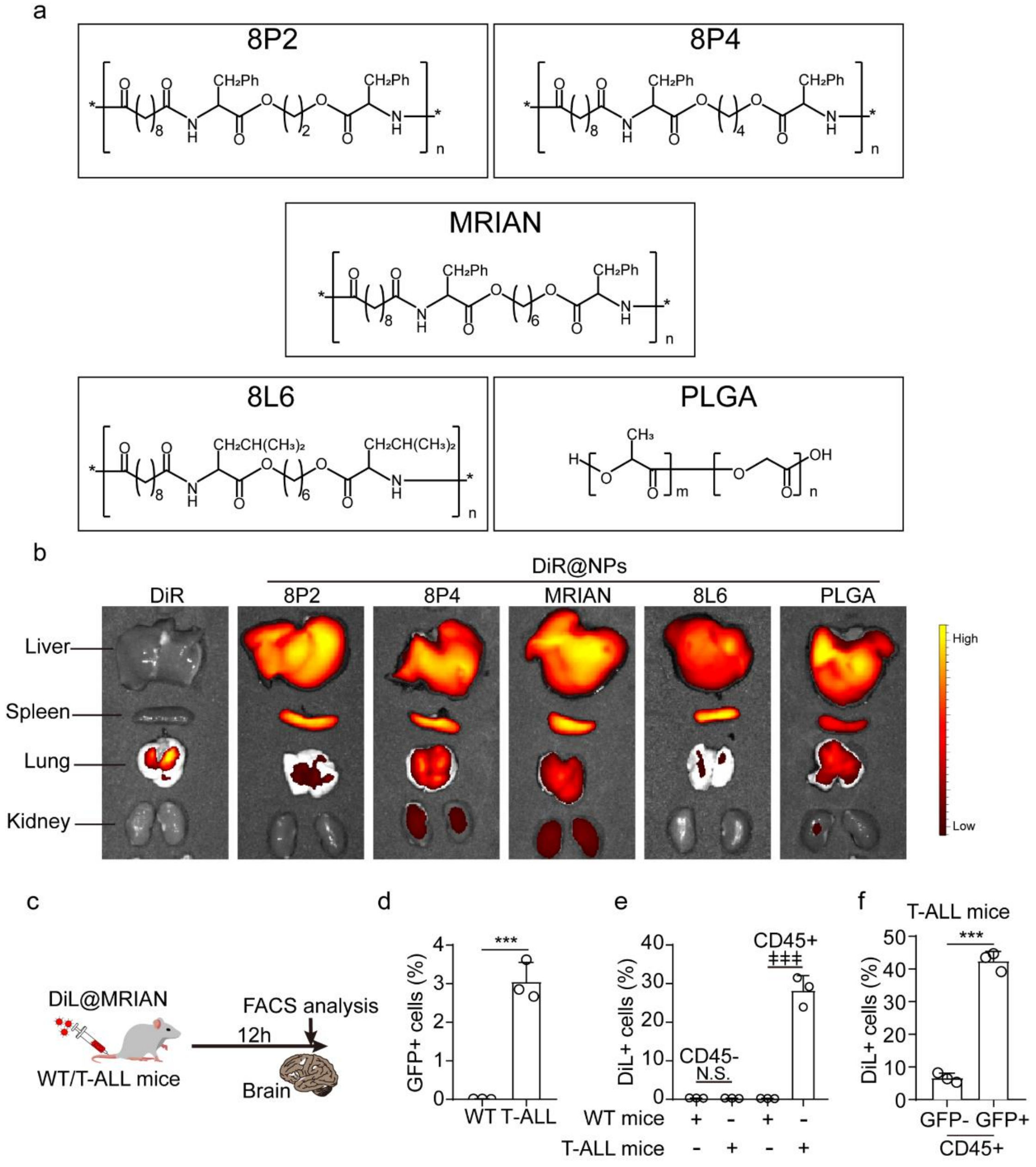

Sup-Figure 1. The structure and tissue distribution of MRIAN and other amino acid-based nanocarriers in T-ALL mice.

(a) Chemical structures for 8P2, 8P4, MRIAN, 8L6, and PLGA.
(b) Bioluminescence images of the liver, spleen, lung, and kidney in T-ALL mice 48 h after NP injection.
(c) Treatment Scheme for T-ALL mice.
(d-f) The frequency of GFP^+ T-ALL cells (d) and DiL^+ cells (e-f) in WT or T-ALL mice brain as indicated.
Data represent mean ± s.d. Two-tailed Student's t-tests were used to assess statistical significance. *P<0.05, **P<0.01, ***P<0.001. Repeated-measures one-way analysis of variance (ANOVA) followed by Dunnett's test for multiple comparisons, ǂ P<0.05, ǂǂ P<0.01, ǂǂǂ P<0.001.

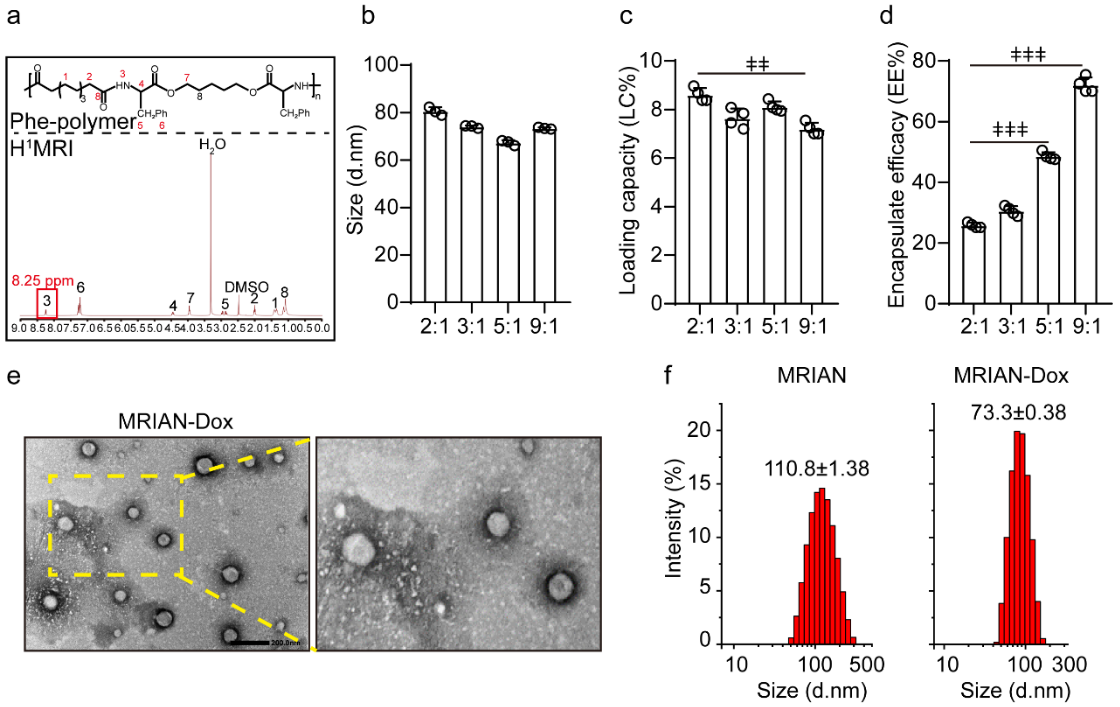

Sup-Figure 2. Synthesis and characterization of MRIAN-Dox.

(a) ^{1}H NMR spectrum of Phe-polymer.
(b-d) The size, LC, and EE of MRIAN.
(e-f) The TEM images (e) and the size distribution (f) of MRIAN-Dox.
Repeated-measures one-way analysis of variance (ANOVA) followed by Dunnett's test for multiple comparisons, ǂ P<0.05, ǂǂ P<0.01, ǂǂǂ P<0.001.

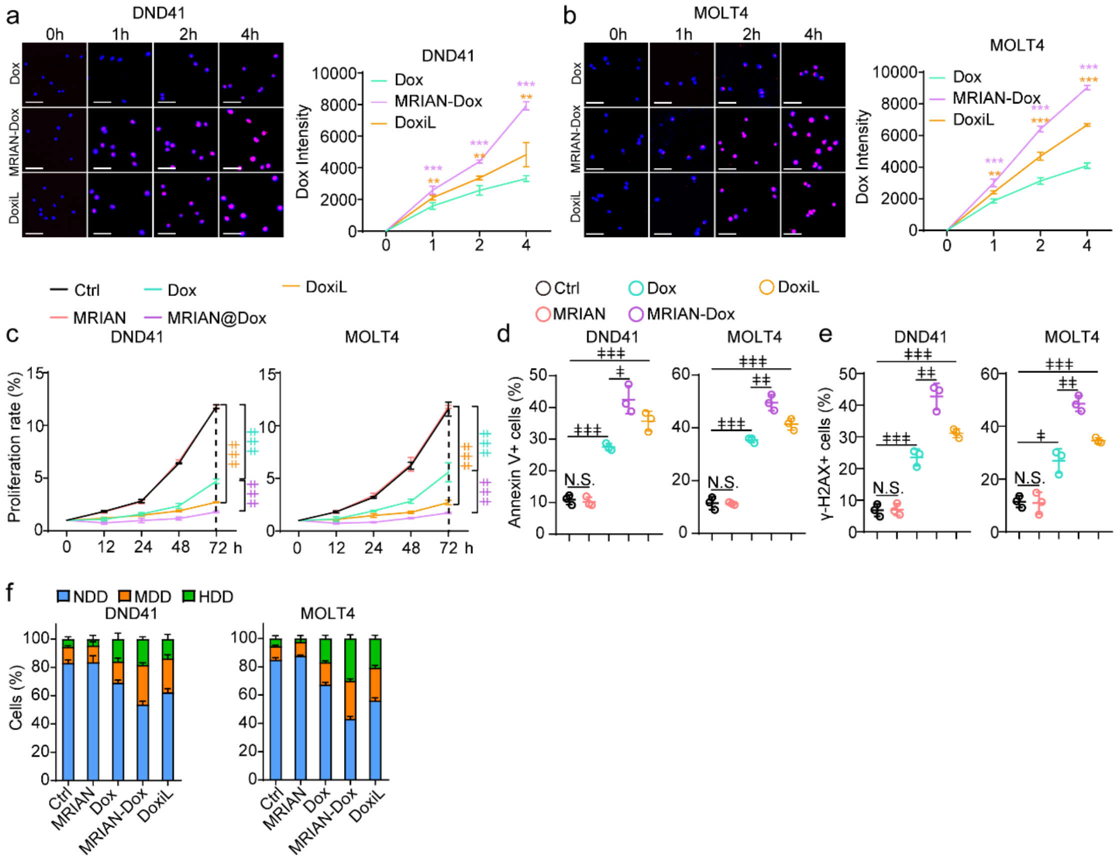

Sup-Figure 3. MRIAN-Dox has enhanced DNA damage- and apoptosis-inducing effects in DND41 and MOLT4 cells.

(a-b) Representative images (left) and quantification (right) of cellular uptake of Dox concentration in DND41 cell (a) and MOLT4 cells (b) after Dox, MRIAN-Dox, and DoxiL treatment as indicated (n=3 independent experiments per group).
(c) Cell proliferation rate of DND41 cells (left) and MOLT4 cells (right) at the indicated time after treatments (n=3 independent experiments per group).
(d-e) Quantification of Annexin V^{+} apoptotic cells (d) and γ-H_2AX^{+} cells (e) in DND41 cells and MOLT4 cells at 72 h after indicated treatments (n=3 independent experiments per group).
(f) Comet assay quantification of DND41 cells and MOLT4 cells at 72 h after indicated treatments (n=50 cells).
Scale bar 50 µm (a and b). Data represent mean ± s.d. Repeated-measures one-way analysis of variance (ANOVA) followed by Dunnett's test for multiple comparisons, ǂ P<0.05, ǂǂ P<0.01, ǂǂǂ P<0.001.

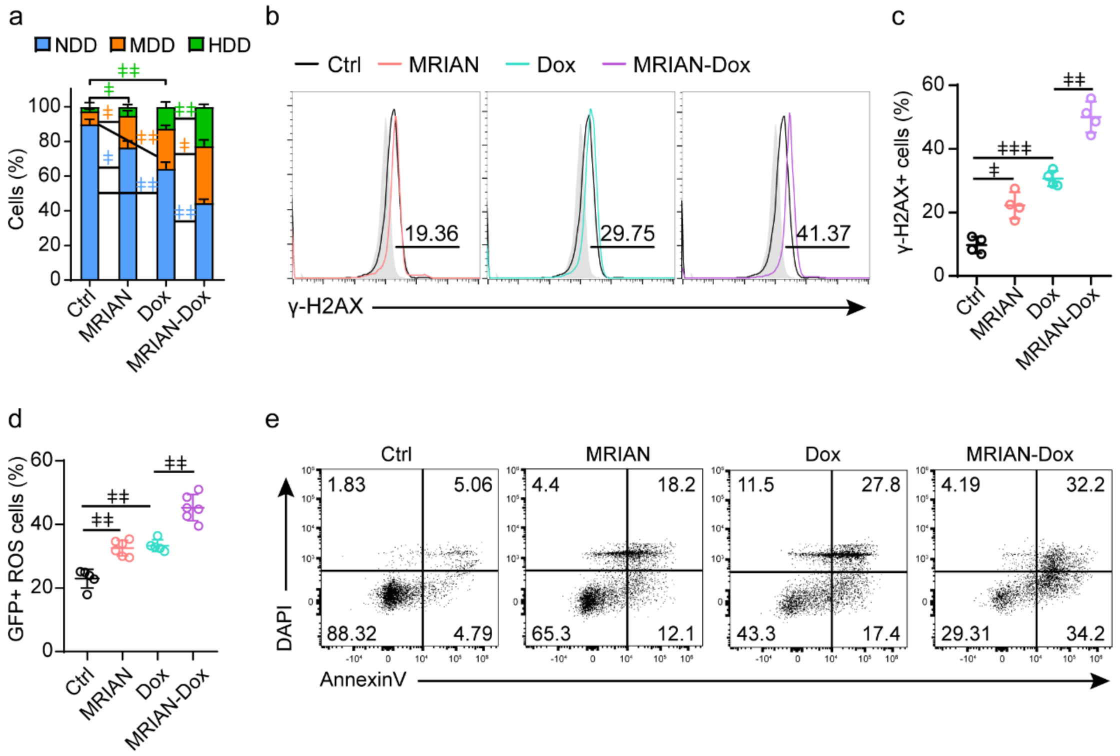

Sup-Figure 4. MRIAN-Dox has enhanced DNA damage- and apoptosis-inducing effects for T-ALL cells in T-ALL mice

(a) Quantification of comet assay of leukemia cells from T-ALL mice after indicated treatment (n=50 cells).
(b and c) Representative γ-H_2AX FACS Histogram (b) and quantification (c) of leukemia cells from T-ALL mice after indicated treatment (n=4 mice per group).
(d) Quantification of ROS level of leukemia cells from T-ALL mice after indicated treatment (n=6 mice per group).
(e) Representative Annexin V FACS plots of leukemia cells from T-ALL mice after indicated treatment (n=4 mice per group).
Data represent mean ± s.d. Repeated-measures one-way analysis of variance (ANOVA) followed by Dunnett's test for multiple comparisons, ǂ $P<0.05$, ǂǂ $P<0.01$, ǂǂǂ $P<0.001$.

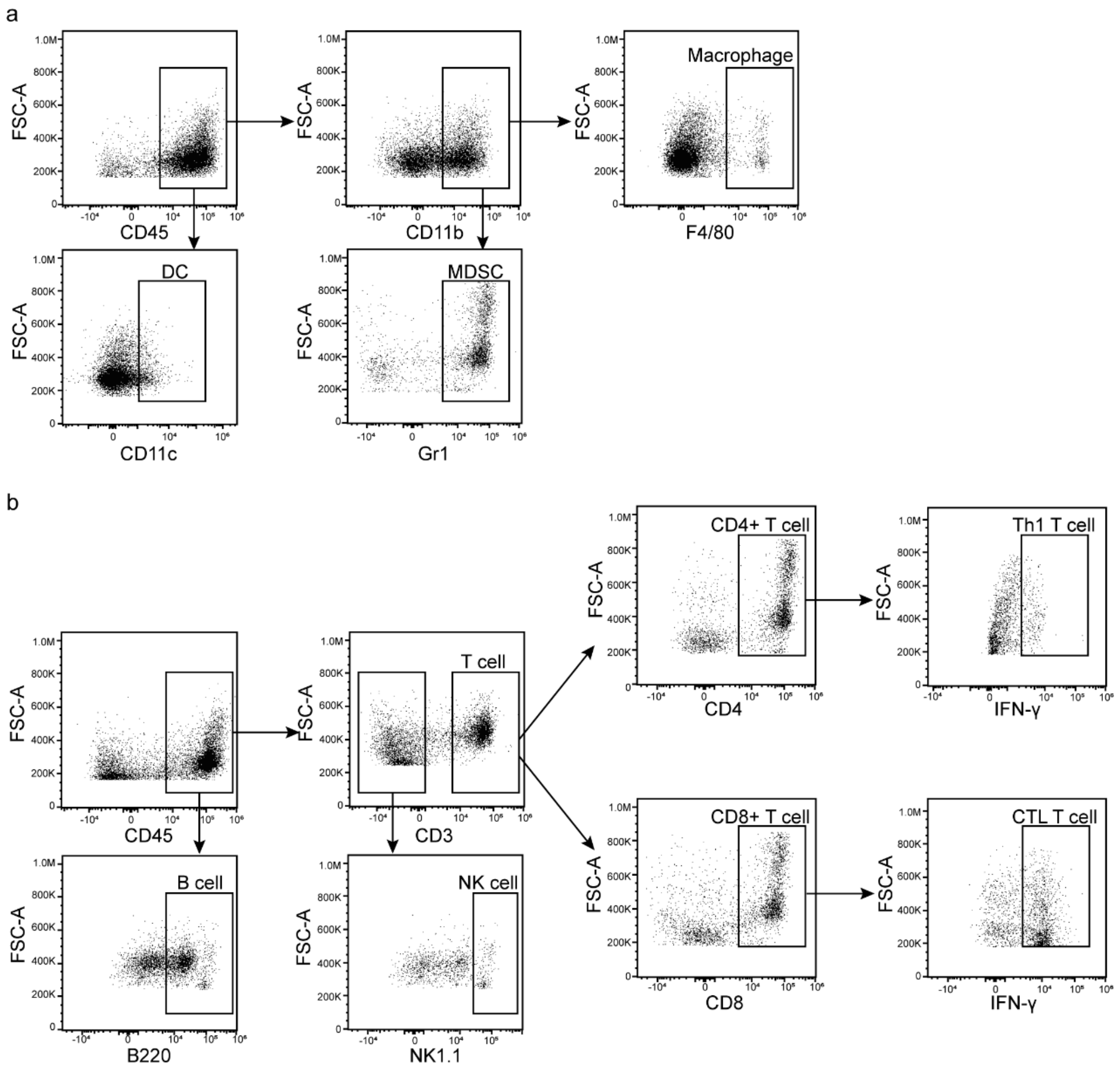

Sup-Figure 5. MRIAN-Dox regulate MDSCs differentiation in vitro

(a-b) Gating strategy in T-ALL BM cells.
